# Technology for fostering intergenerational connectivity: scoping review protocol

**DOI:** 10.1186/s13643-017-0652-y

**Published:** 2017-12-08

**Authors:** Jennifer Boger, Kathryn Mercer

**Affiliations:** 1 Department of Systems Design Engineering, University of Waterloo, 200 University Ave., Waterloo, ON N2L 3G1 Canada; 2 Research Institute for Aging, Waterloo, ON Canada; 3 University of Waterloo Library, University of Waterloo, Waterloo, ON Canada; 4 School of Pharmacy, University of Waterloo, Waterloo, ON Canada

**Keywords:** Scoping review, Technology, Older adults, Elderly, Youth, Children, Intergenerational connectivity, Engagement, Connecting generations

## Abstract

**Background:**

The simultaneous increase in geographically dispersed families and general decrease in engagement in local communities is resulting in fewer opportunities for youth and older adults interact in meaningful ways. Technology is becoming increasingly pervasive and flexible and providing new opportunities to foster intergenerational connection that can be implemented and evaluated across a multitude of populations and contexts. What research has been done in this area is spread across disciplines and what aspects of technologies could make them more effective is not well understood.

**Method:**

The scoping review will be completed in five stages: (1) identifying the research question, (2) identifying relevant studies, (3) selecting studies, (4) charting the data, and (5) collating, summarizing, and reporting the results. Comprehensive descriptive data from each study will be presented along with an analysis of similarities and differences in research from different disciplines.

**Discussion:**

This scoping review focuses on a search of the literature to gain an understanding of what technologies have been used specifically for fostering intergenerational connectivity and to establish what future directions for research could be. To the authors’ knowledge, it is the first scoping review of its kind.

**Electronic supplementary material:**

The online version of this article (10.1186/s13643-017-0652-y) contains supplementary material, which is available to authorized users.

## Background

Social interactions lie at the heart of a functional society. Interactions between the youth (people younger than 19 years old) and older adults (people over 50 years old) provide a natural and socially healthy mechanism for the mutual exchange of knowledge, values and skills and have been shown to significantly benefit both populations. The literature review conducted by Springate and colleagues [1] concludes that effective intergenerational programming had positive outcomes related to health and well-being, isolation and sense of worth for older adults, gaining of specific skills and self-esteem for younger adults, and increased understanding, friendship, enjoyment and confidence for both groups. This is supported by research such as the intergenerational mentoring program developed by Au et al. [2], where university students provided 40 h of volunteer support to frail older adults over a 4-month period. This interaction resulted in significant and positive changes in meaning of life, service motivation and ageism for the youth. Marcia et al. [3] demonstrated that a monthly vising program to an assisted living facility by elementary students improved self-perception and mitigated behavioural difficulties in the classroom; the program was considered to be enjoyable for both the younger and older adults. Kemp’s work with grandparent-adult grandchildren demonstrated that ongoing relationships provide a “safety net” for one another, particularly regarding social and emotional support [4].

While there are clear advantages to intergenerational connectivity, global society is increasingly shifting toward geographically dispersed parent-and-child(ren) families [5, 6]. Recent social culture trends, particularly in more developed economies, have led to a decrease in connectivity and interactions between members of communities [7–9]. This decrease in interactive opportunities between older adults and youth in families and the community has led to a considerable decrease in connectivity between the older and younger generations [10, 11]. At the same time, lower birth rates and increased life spans means the average age of our global society is expected to increase rapidly; people over the age of 65 are estimated to increase from 8.5% in 2015 to 16.7% in 2050 [12]. Population estimates are higher for developed regions with 27% of Europeans and 21.4% of North Americans over the age of 65 by 2050 [12].

This unprecedented decrease in the ratio of young to old will undoubtedly bring about many societal changes. The benefits of intergenerational connectivity provide mutually beneficial opportunities that inherently mitigate substantial social pressures that may arise from aging populations. For example, Harley et al. discuss how internet, mobile and pervasive technologies explore key themes with different communication issues in an intergenerational context, and technologies are emerging as a meaningful way of social engagement [13, 14]. Another example is that reciprocal volunteerism between the young and old would enable access to mutually beneficial skills or resources while simultaneously providing potential opportunities for generationally situated information, longer-term intergenerational relationships, and reduced demands on formal service providers. Moreover, increased lifespans mean that generations will concurrently age for longer than ever before, resulting in longer periods of time to form and benefit from meaningful relationships [4].

Intergenerational connectivity may provide mutually beneficial opportunities that mitigate social pressures arising from aging populations. Four in 10 older adults now own smartphones and, as a group, are increasingly digitally connected, owning and adopting technologies at a similar rate to those under 65, with similar increasing trends in uptakes of social media [15]. While older adults face different barriers to uptake, including physical challenges alongside lack of confidence and awareness, research has shown that older adults are likely to seek help in how to understand and use technologies [15]. The increasingly ubiquitous presence of technology in society offers an opportunity for connecting youth and older adults in new and innovative ways. Interventions could be implemented and evaluated across a multitude of population characteristics such as age, socioeconomic backgrounds, health, geography, and technology characteristics such as type, purpose and format. While technologies are being created with this goal in mind, there is still much we need to understand in order to engage in this area of research in a thoughtful and impactful way. By identifying what has been done, we may understand what has been effective, what could be improved and what gaps exist.

Despite there being significant research on aging, intergenerational partnerships and technology, it is not yet clear to the extent what empirical research has been done that involves all three aspects. To the authors’ knowledge, there is no published synthesis on technologies in general that have been developed or used with the specific intention of fostering intergenerational connectivity.

The primary goal of this scoping review is to identify the current scope of research regarding technologies that have been specifically designed or used to foster intergenerational connectivity. To achieve this, we will identify characteristics of the research and related technologies, map key insights from these into thematic domains and determine how these data can be used to target future research efforts. This work constitutes the first step of a research program intended to develop evidence-based technology and programs that can foster intergenerational connectivity; insights from conducting this review will be used to guide future research. It is anticipated that this review will support researchers and others in the assessment of interventions that use existing technology as well as the formulation of new technologies to foster intergenerational connectivity.

## Methods/design

The research team for this review has extensive experience in the areas of technology, aging, intergenerational communities, health, biomedical engineering and information studies.

### Protocol development

This scoping review will follow the methodology established by Arskey and O’Malley and adapted by Levac et al. [16, 17]. The stages include (1) identifying the research question, (2) identifying relevant studies, (3) study selection, (4) charting the data, and (5) collating, summarizing and reporting the results. The PRISMA-P checklist is included as an additional file [see Additional file 1]. PROSPERO registration is not required as it is a scoping review.

#### Stage 1: Identifying the research question

Research regarding technology for connecting older adults and youth has been discipline-centric (e.g. computer science and sociology) with minimal crossover; this has resulted in the research in this area being scattered, with interventions focusing on aspects that align (often solely) with the developer(s)’ specific discipline. Moreover, while some systematic, scoping and literature reviews regarding using technology to connect generations have been conducted, they have focused on a specific aspect, such as technologies for encouraging game play between the generations [18, 19], development of intergenerational programs that use technology [20] or older adults’ use of ambient and networking technologies [21].

The lack of a comprehensive overview that includes all disciplines led to the guiding research question for this scoping review, which is “What technological interventions exist that are specifically designed to foster inter-generational connectivity?”

#### Stage 2: Identifying relevant studies/search strategy

ACM, IEEE, MEDLINE (PubMed), CINAHL, Scopus and Web of Science databases will be searched for relevant publications using predefined search terms. These databases were selected to capture the diversity of disciplines where relevant work is likely to be indexed and reflect the potential scope of relevant literature.

Keywords used for search terms will include: older adult*, social media, social network, adolescent*, aged, application, app, child*, computer, communication*, community connectivity, cross-generational, device, digital, elder*, geriatric, grandchild*, grandparent*, intergeneration*, intervention*, learning, mentoring, outreach, partnership, program, relations, senior*, senior citizen, software, student, teaching, technolog*, telehealth, youth*.

The research team will use a three-stage approach to identify appropriate publications. The first stage is searching the six electronic databases using standardized search terms adapted to the requirements of each respective database. The second stage involves searching the reference lists of literature that meets all inclusion criteria (outlined in the “Eligibility criteria” section below). The third and final stage involves hand searching specific key publications such as identified white papers or conference presentations for any references we may have missed.

#### Stage 3: Study selection

##### Type of studies

The goal of this review is to determine the amount, type, and focus of existing peer-reviewed research that has been done (i.e. peer-reviewed journal papers and conference papers that employ any different methodology). Grey literature (e.g. conference presentations, white papers, theses and research reports) will not be included. Searches will not be limited by a date range.

Studies will be considered eligible if they address aspects of creating or using technologies with the specific intent of building intergenerational connections, in person or virtually (e.g. over the internet), through interaction of youth and older adults. Secondary use of technologies, such as using a car for a younger person to visit an older one, will not be included. Example topics that would be eligible for inclusion are:

- Grandchild(ren)/grandparent(s)’ views and experiences regarding shared online communities
- Technology-based interventions designed to facilitate intergenerational knowledge exchange
- Online partnerships in sharing or learning about culture
- Development of technology meant to connect and foster intergenerational leisure activities

We will be including all studies regardless of methodology or design to provide a rich and descriptive narrative of the current state of research on intergenerational connectivity, while acknowledging that as an emerging field there is no existing research cohesion.

##### Eligibility criteria

The following inclusion criteria will be used:

- Published in the English language
- Must contain both youth below 21 and adults over 50 years of age
- Peer-reviewed primary research (e.g. journal and conference publications)
- Design or use of a technology with the specific intention of fostering intergenerational connectivity

Exclusion criteria are:

- Publications that are not peer-reviewed reviewed, such as editorials, book reviews, commentaries or opinion pieces
- Programs or interventions where the design or use of technology was not a focus (e.g. summer camps or mixed residences)
- Literature, scoping, systematic and other reviews

While review articles (i.e. scoping reviews, systematic reviews, meta-analyses, narrative reviews) will not be included in in the scoping results, they may be used in the background and/or discussion.

We will consider a broad range of digital technologies used to support connectivity, including apps, wearable fitness, online tools, and custom-built applications. We are not defining any geographic, socioeconomic, or health inclusion or exclusion criteria.

##### Selection of items for inclusion

Publications will be selected and screened using a three-stage process. The first stage is to use the inclusion and exclusion criteria (described above) to select possible studies for inclusion based on returned titles from database searches. The second stage will involve screening the abstracts of the publications identified in stage 1 using the same criteria. The third and final stage will be a full text read-through of papers that passed the first two stages. All stages will be performed by two independent reviewers. Any disagreements regarding inclusion at any of the stages will be resolved by a third party and, if necessary, face-to-face discussion by the review team. The selection and exclusion process will be organized using a PRISMA flowchart.

#### Stage 4: Charting the data

Data extraction and organization will be performed using a set of standard metrics (e.g. study location, sample size, methodology and discipline) and relevant metrics (e.g. type of technology used and relationship of youth/older adults).

A combination of EndNote X8 and Microsoft Excel 2013 will be used to organize and track relevant data. We will use these software to (1) remove duplicates; (2) document and manage the screening process; (3) categorize publications that meet our inclusion and exclusion criteria; (4) extract, organize, and search related data and information from the publication content and (5) manage of full text versions of included publications, including adding relevant notes that include key data extraction insights.

#### Stage 5: Data synthesis

Identified publications will be described in terms of location where the study was performed; research field(s) of those performing the study; sample size of the study; type/description of technology used; purpose of technology used and study design and methodology. We will present the descriptors as text, tables and graphs. We acknowledge that we may not be able to analyze and report on risk of bias and strength of methodology, quality, rigour and other outcomes due to the wide variety of studies being examined, and because this is an emerging field, there is no standard methodology. We will be guided by the Critical Appraisal Skills Programme tool for qualitative research, the Quality Assessment Tool for Quantitative Studies, and the Mixed Methods Assessment Tool when looking at studies included [22–24].

We will also be conducting a content analysis to identify which aspect(s) of technology the study focuses on, identify emergent themes and collect and identify objectives and gaps in our understanding of the current state of research. The discussion will be structured based on the themes that emerge.

## Discussion

The aim of this review is to provide an overview of peer-reviewed publications related to technology for fostering intergenerational connectivity. Since there is no established vocabulary in this emerging field, the search strategy will likely be iterative in nature to maximize our ability to capture all relevant publications. Unfortunately, this broad searching technique is often associated with a lower precision and a larger number of redundant references; however, most irrelevant publications should be excluded during title and abstract screening.

A careful and focused effort will be put into developing the searches, standardizing our selection and data collection forms through collaboration with an expert librarian. These documents will be established prior to the start of the review and their content and structure thoroughly assessed during pilot-testing. Through the data collection process, there may be refinement of the search terms and protocol to ensure that the quality, consistency, and structure of the data extracted is methodical and appropriate. Additionally, due to the nature of a scoping review on a topic with relatively little written about it, the searches will be more iterative in their nature.

Our methodology is grounded in validated research practices; however, we acknowledge limitations to the review. Limiting included papers to English will result in a language bias. There will also be limitations to publication type as our goal is to scope peer-reviewed literature; therefore, gray literature and other non-peer-reviewed publications and reviews will be excluded. Finally, as this review is intended to achieve a high-level understanding of the state of the field, there will be no formal assessment of study quality.

This study is the first step of a comprehensive research program that will be aimed at developing innovative technologies and systems for fostering intergenerational connectivity. As such the review will aim to (1) identify what peer-reviewed literature exists, (2) summarize relevant publications and (3) discern overreaching themes. These results will be used to target one or more identified key areas to better understand how technology can be used to bring different groups of people closer together, guide best practices in technology design, and inform the development of new technology. The results of this paper will more broadly offer foundational knowledge regarding terminology, methods and existing research that can inform new research, as well as creation of new technologies. Our research team will use an interdisciplinary approach to knowledge translation, including using clear and generalizable vocabulary in the interpretation of the results and presenting findings that will be of interest to more than one discipline.

## Additional file

Additional file 1: PRISMA-P (Preferred Reporting Items for Systematic review and Meta-Analysis Protocols) 2015 checklist: recommended items to address in a systematic review protocol. Description of data: This file is the completed PRISMA-P 2015 checklist for items to address in a systematic review protocol for the “Technology for fostering intergenerational connectivity” scoping review protocol. (PDF 54 kb)
